# Oxidative Activity of Copper(II) Complexes with Aminoglycoside Antibiotics as Implication to the Toxicity of These Drugs

**DOI:** 10.1155/S1565363304000056

**Published:** 2004

**Authors:** Wojciech Szczepanik, Piotr Kaczmarek, Małgorzata Jeżowska-Bojczuk

**Affiliations:** Faculty of Chemistry University of Wrocław F. Joliot-Curie 14 Wrocław 50-383 Poland

## Abstract

The majority of aminoglycosidic antibiotics anchor Cu(ll) ions by {NH_2_, O} chelates of the A and C rings of its molecule as distinct from amikacin, which belongs to the class of substituted ones. The results indicate that all these antibiotics effectively bind copper(ll) at physiological pH. Cyclic voltammetry investigations and kinetic studies of H_2_o_2_ disproportionation and hydroxyl radicals detection made it possible to support the mechanism of oxidative reactivity of cupric complexes of aminoglycosides, which involves Cu(1) and Cu(lll) redox states and metal-bound, rather than free radical species. The mechanism of this process appears to be complicated, and may have deleterious side-effects by leaking radical intermediates. The presence of these reactive oxygen species may be responsible for modulating the biological activity of these drugs.

The interactions of copper(ll) complexes of aminoglycosides with oxidation-susceptible biomolecules: 2’-deoxyguanosine, plasmid DNA and yeast tRNA^phe^ in both the presence and absence of hydrogen peroxide showed that the complexes with H_2_o_2_ are the most efficient oxidants, converting dG to its 8-oxo derivative, generating strand breaks in plasmid DNA and multiple cleavages in tRNA^phe^. Some of these reactions may play a role in aminoglycoside-induced ototoxicity and nephrotoxicity; moreover, they may suggest that Cu(ll)-aminoglycosides are potentially dangerous genotoxic agents. These complexes were also screened for their antibacterial activity and bioassays were engaged to find out the possibility of Cu(ll)-kanamycin A complexes to induce tumor necrosis factor (TNF), interferon (IFN) and interleukin-10 (IL-10) in human peripheral blood leukocytes. The aim of these studies was to compare the biological action of antibiotic alone and complexed with copper(ll) ions in both neutral and oxidative environment.


### Oxidative Activity of Copper(II) Complexes with Aminoglycoside Antibiotics as Implication to the Toxicity of These Drugs

Wojciech Szczepanik[†], Piotr Kaczmarek and Małgorzata Jeżowska-Bojczuk*

*Faculty of Chemistry, University of Wrocław, F. Joliot-Curie 14, 50-383 Wrocław Poland*
**e-mail: mjb@wchuwr.chem.uni.wroc.pl. Tel: +48-71-3757281, Fax: +48-71-3282348*
*[†]Supported by The Foundation for Polish Science*

**ABSTRACT**

The majority of aminoglycosidic antibiotics anchor Cu(II) ions by $\{NH_2, O^-\}$ chelates of the A and C rings of its molecule as distinct from amikacin, which belongs to the class of substituted ones. The results indicate that all these antibiotics effectively bind copper(II) at physiological pH. Cyclic voltammetry investigations and kinetic studies of $H_2O_2$ disproportionation and hydroxyl radicals detection made it possible to support the mechanism of oxidative reactivity of cupric complexes of aminoglycosides, which involves Cu(I) and Cu(III) redox states and metal-bound, rather than free radical species. The mechanism of this process appears to be complicated, and may have deleterious side-effects by leaking radical intermediates. The presence of these reactive oxygen species may be responsible for modulating the biological activity of these drugs.

The interactions of copper(II) complexes of aminoglycosides with oxidation-susceptible biomolecules: 2'-deoxyguanosine, plasmid DNA and yeast $tRNA^{Phe}$ in both the presence and absence of hydrogen peroxide showed that the complexes with $H_2O_2$ are the most efficient oxidants, converting dG to its 8-oxo derivative, generating strand breaks in plasmid DNA and multiple cleavages in $tRNA^{Phe}$. Some of these reactions may play a role in aminoglycoside-induced ototoxicity and nephrotoxicity; moreover, they may suggest that Cu(II)-aminoglycosides are potentially dangerous genotoxic agents.

These complexes were also screened for their antibacterial activity and bioassays were engaged to find out the possibility of Cu(II)-kanamycin A complexes to induce tumor necrosis factor (TNF), interferon (IFN) and interleukin-10 (IL-10) in human peripheral blood leukocytes. The aim of these studies was to compare the biological action of antibiotic alone and complexed with copper(II) ions in both neutral and oxidative environment.

**Keywords** Aminoglycoside antibiotics, cupric complexes, hydroxyl radicals, DNA damage, RNA hydrolysis, antibacterial activity, cytokines

**Abbreviations:** NDMA – N,N-dimethyl-p-nitrosoaniline, NBT – Nitro Blue Tetrazolium, DMPO – 5,5-dimethyl-1-pyrroline-N-oxide, ROS – reactive oxygen species, dG – 2'-deoxyguanosine, 8-oxo-dG – 7,8-dihydro-8-oxo-2'-deoxyguanosine, Y37 – wybutosine in position 37 of $tRNA^{Phe}$, IFN – interferon, IL-10 – interleukin 10, TNF – tumor necrosis factor.

### INTRODUCTION

Aminoglycoside antibiotics constitute a very important class of antibiotics, active against Gram-negative and some Gram-positive bacteria. The mechanism of their action has been studied very extensively since the discovery of their first representative – streptomycin. Their action is based on the interactions with ribosomal RNA and also cytoplasmic membrane /1/. Apart from ribosomal RNA these drugs were proved to interfere with HIV-1 viral Rev responsible element /2/ and its different constructs /3/ as well as TAR RNA /4/, group I introns /5/, hammerhead ribozyme /6/, RNase P RNA /7/ and yeast $tRNA^{Phe}$ /8/. The current knowledge of the mode of action of aminoglycosides reports several adverse effects, such as ribosomal blockage, misreading in translation, cellular membrane damage and irreversible uptake of the antibiotic /9/. Aminoglycosides are still problem antibiotics since their therapy requires constant monitoring of the drug concentration in blood plasma. The reason for this caution is a coexistence of various toxic effects such as ototoxicity and nephrotoxicity /10/. Moreover, this group of antibiotics became suspected of another set of deleterious side effects: hemato- , hepato- and neurotoxicity. It reveals a serious demand for the improved and modified ones, but also for the detailed look into the chemical basis of their action. The drastic toxicity of aminoglycosides, however widely explored, is still unclear. The formation of reactive oxygen species was postulated to accompany the application of these drugs. Fe(II) complexation and generation of hydroxyl radicals by gentamicin /11, 12/ and neomycin /13/ has recently been implicated in the mechanism of its toxicity. As aminoglycosides themselves are redox-inactive, they need a specific medium to participate in the oxidative pathway they undergo in human organism. Metal ions seem to be suspected the most. Literature includes only few reports concerning comparative coordination study of some transition metal ions like Co(II), Ni(II), Zn(II) and Cu(II) to aminoglycosides /14, 15/, as well as antimicrobial activity experiments of those complexes /16/. Our recent investigations in this area demonstrate that these drugs, *i.e.* kanamycin A, bind copper(II) several hundred fold stronger than other metal ions, both essential and toxic /17/. This fact prompted us to undertake a profound study on Cu(II)-aminoglycosides coordination patterns and mechanisms of reactivity of the resulting complexes. However, the amount of "free" Cu(II) in organism is low. Moreover, intracellular copper is under very tight control, exerted by specific chaperone proteins /18/. Extracellular copper is not controlled as tightly and is elevated in some pathological conditions, including cancer and inflammation /19/. It is possible then, that some copper may be transferred to the molecule of antibiotic, which gave us the basis for our investigations.

### COPPER(II) COMPLEXATION BY AMINOGLYCOSIDES

Aminoglycosides possess a few donating groups in their molecules. These are mainly amino functions, whose number varies between three and six. All these amines coexist with hydroxyl groups in their neighbourhood. Such combinations of donors provide strong chelating sites for many biologically accessible metal ions. The results of our studies provided evidence that from a range of metallic microelements, like copper, iron, zinc, cobalt or nickel, aminoglycosides form the most stable complexes with Cu(II) ions /17/.

The binding modes of the cupric complexes of unsubstituted aminoglycosidic antibiotics, like kanamycins, involve both terminal aminosugar rings, chelating central metal ion by amine and hydroxyl functions /20-24/ (Fig. 1). Distinctly different is the coordination pattern of copper(II) complexes of amikacin /25/ (Fig. 2). This antibiotic is a modified version of kanamycin A, having one amine group acylated. The new formed amide function, together with neighbouring amino group constitute very strong binding site for copper(II) and coordinate the metal ion at physiological pH with a peptide fashion. The presence of Cu(II) ion enforces the specific, coiled structure of the complex molecule, what could be of significant importance for the biological activity of the antibiotic. To characterize the complex species potentiometry was used, to provide the stability constants and stoichiometry of the complexes. Spectroscopic methods (electronic absorption, EPR and CD) brought evidence for the nature of donating groups and mass spectrometry helped to confirm the stoichiometries of the species in solution.

It was shown that bleomycin can sequester copper(II) and introduce it into the cell /26, 27/. Such an effect on the metal homeostasis might also accompany the therapy with use of aminoglycosides. In order to examine whether the antibiotic could be capable of disturbing copper(II) content in blood plasma, the ability of kanamycin A to remove Cu(II) ions from saturated N-terminal binding site of human serum albumin was tested /17/. The formation of $NH_2 \rightarrow Cu(II)$ charge transfer transition on CD spectra, characteristic for copper(II) complexes of aminosugar-like ligands /20-23/ was observed. This would suggest that at favourable conditions the complex formation might be possible, especially in case of high antibiotic concentration in plasma, which may occur during intravenous or intramuscular injection.

### OXIDATIVE ACTION OF Cu(II)-AMINOGLYCOSIDES

The oxidative properties of copper centres in metalloproteins are well known. Many cupric complexes of xenobiotics were also proved to induce processes involving reactive oxygen species. Following that we undertook studies concerning the oxidative action of the copper(II) complexes of aminoglycosidic antibiotics. The experiments were planned to characterize the possible oxidation states of copper centre, the behaviour of the complexes in the presence of physiological amounts of hydrogen peroxide as well as description of the reactive intermediates of the reactions and their final products.

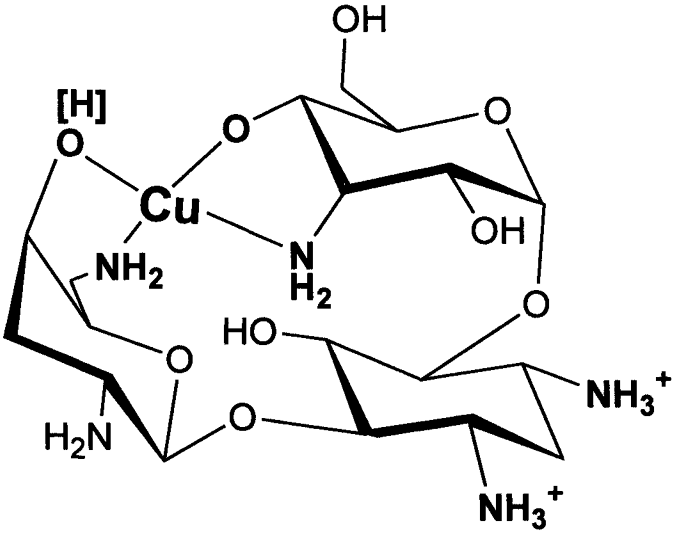

**Fig. 1:** The Cu(II) complex of kanamycin A, predominating around physiological pH.

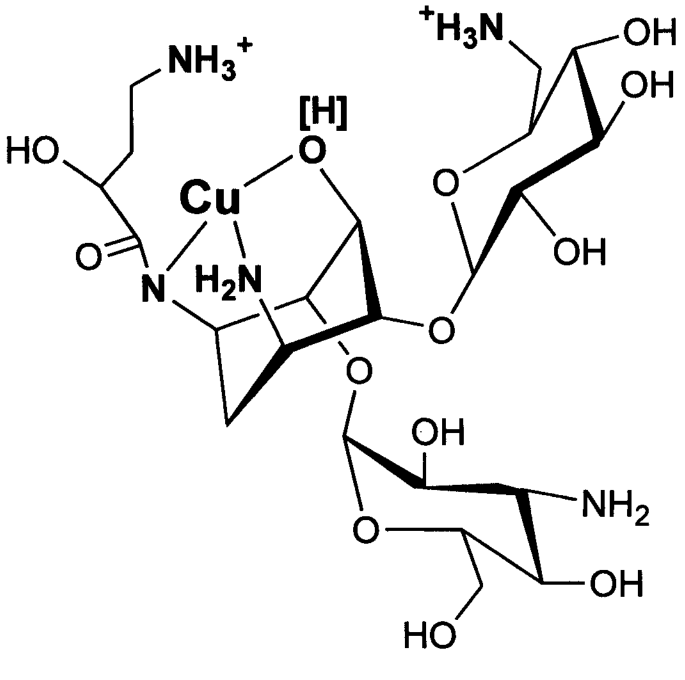

**Fig. 2:** The Cu(II) complex of amikacin, predominating around physiological pH.

To identify possible radicals generated from $H_2O_2$ by Cu(II) complexes of aminoglycosides we performed the experiments with two relatively specific reporter molecules: NBT, which reacts with superoxide radicals, and NDMA, which is commonly regarded as specific towards hydroxyl radicals. Only Cu(II)-kasugamycin complex was capable of superoxide radicals production /28/. Kasugamycin is not a typical aminoglycoside and its copper chelating fashion is distinct from that observed for other antibiotics used in our study. The classic aminoglycosides failed to demonstrate superoxide formation. However, the decomposition of NDMA in their case presented the formation of strongly oxidizing ROS following $H_2O_2$ addition /23, 29/. Figure 3 shows the decrease of the characteristic band for NDMA absorption at 440 nm. This process is the most effective in the presence of the complex and hydrogen peroxide, while uncomplexed copper induces ROS production not so effectively. To carry out the unambiguous identification of the radical species, EPR experiments were performed in the presence of DMPO. They yielded spectra characteristic for the adduct with hydroxyl radical. Addition of excess ethanol to the system resulted in a complete quenching of radical formation (Fig. 4).

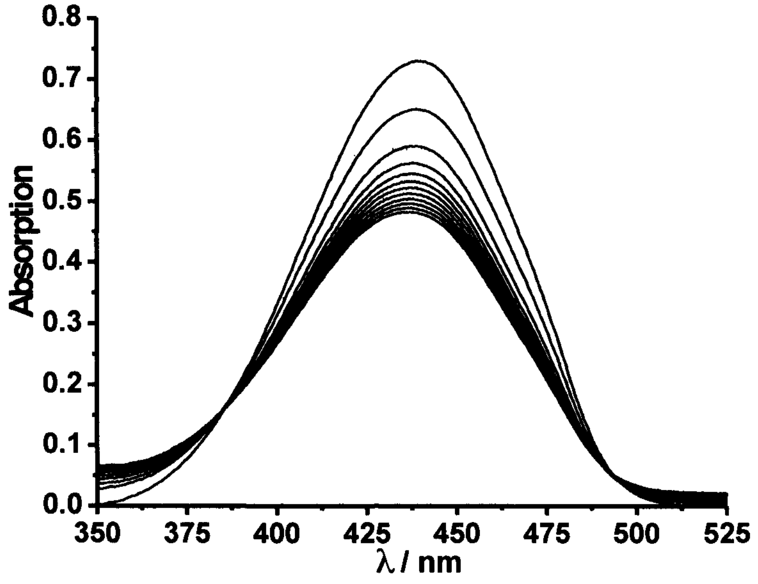

**Fig. 3:** The decrease of the NDMA characteristic band at 440 nm induced by Cu(II)-aminoglycoside-$H_2O_2$ system at pH 7.4.

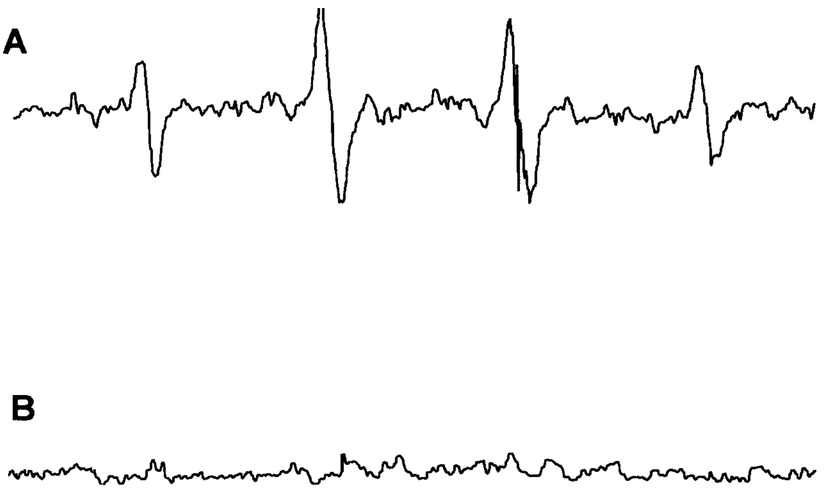

**Fig. 4:** **A.** The characteristic EPR spectra of •OH adduct with DMPO in the presence of Cu(II)-aminoglycoside-$H_2O_2$ system at pH 7.4. **B.** The spectra quenching after addition of ethanol to the solution.

The comparative experiment performed for the selected antibiotics showed that the most active is the cupric complex of neomycin B /30/. It has also some reflection in toxicology. Beside streptomycin, which was not included in the study due to its tendency to hydrolyse in the presence of metal ions, neomycin and kanamycin B are the most toxic aminoglycosides to the kidney and inner ear and thus their clinical usage has been limited. This process exhibits the distinct pH dependence /23/. The diagram at Figure 5 indicates that complex in which maximum concentration exists around physiological pH is the most efficient radicals producer. The pH profile of the control samples, containing uncomplexed Cu(II) ions, is quite different, with a maximum at pH 9 – where soluble Cu(II) hydroxides predominate over insoluble ones.

Cyclic voltammetry studies indicated that kanamycin A and amikacin are redox-inactive, while their copper(II) complex exhibited irreversible reduction and oxidation peaks in a wide pH range /23, 28/. The cathodic scans revealed the presence of copper(III) ions in the complex. However, gasometric measurements showed that these complexes not only activate hydrogen peroxide but also disproportionate it to water and dioxygen, which are the final products of the process. Quantitative description of oxygen generation phenomenon, including the presence of Cu(III) ions and hydroxyl radicals as intermediate products, led us to evaluate the mechanistic considerations /23, 28/:

$$Cu(II)\text{-}L + H_2O_2 = Cu(I)\text{-}L\text{-}^{\bullet}OOH + H^+$$

$$Cu(II)\text{-}L + H_2O_2 = Cu(III)\text{-}L\text{-}^{\bullet}OH + OH^-$$

$$Cu(I)\text{-}L\text{-}^{\bullet}OOH + Cu(III)\text{-}L\text{-}^{\bullet}OH = 2\ Cu(II)\text{-}L + O_2 + H_2O$$

$$2\ H_2O_2 = O_2 + 2\ H_2O \text{ (sum reaction)}$$

where L means the ligand molecule. The hydroxo- and hydroperoxo- forms of the antibiotic molecules accompany these reactions. Their presence was confirmed by mass spectrometry, which showed that the initial target of oxidations is the complexed aminoglycoside molecule /31/. Apart from their formation, fragmentations at glycosidic bonds and in the amide bond, linking the aglycon to ring B, were detected (Fig. 6).

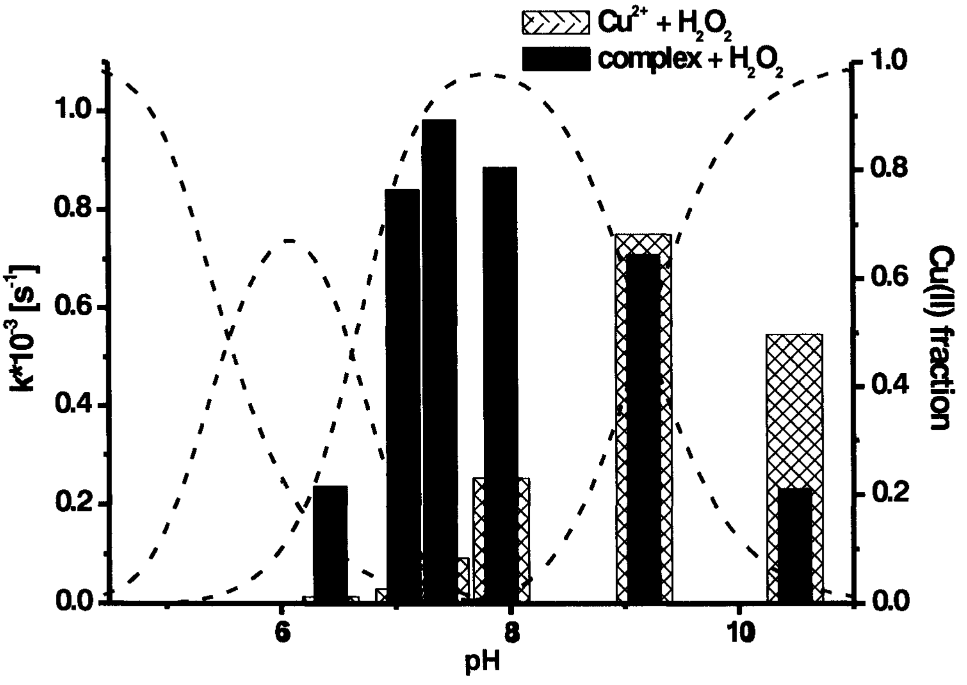

Fig. 5: The comparison of pH influence on the rates of NDMA detected hydroxyl radicals formation by Cu(II)-kanamycin A complex and Cu(II) ions, both in $H_2O_2$, overlaid with the species distribution diagram for Cu(II) ions and antibiotic.

### INTERACTIONS WITH NUCLEIC ACIDS AND COMPONENTS

Another type of oxidative activity engaging ROS, regarded in addition as a test for procarcinogenic or promutagenic properties of the compound studied, is 2'-deoxyguanosine oxidation /31, 32/. Production of 8-oxo-guanosine suggests that these complexes may oxidize nucleobases within DNA molecules and the consecutive impairment of bases may lead to deleterious results, like mutations. This process seems to be pH dependent, showing thereby that the complex present around physiological pH is the most efficient 2'-dG oxidizing agent /32/ (Fig. 7). The same species are also responsible for the specific 2'-dG conversion to its 8-oxo derivative.

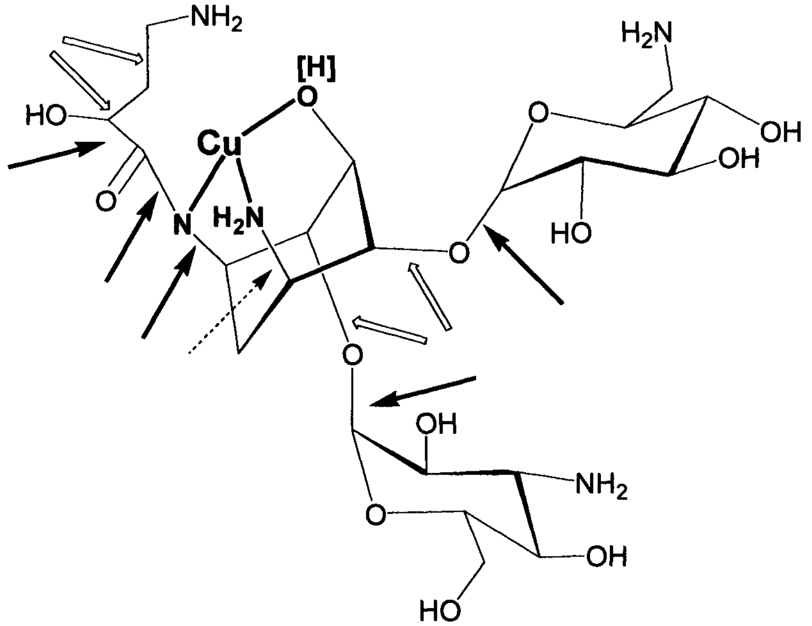

**Fig. 6:** The schematic drawing of the Cu(II)-amikacin complex at pH 7.4, with the sites of breakage indicated by ESI-MS and MS/MS experiments. Arrows indicate the positions of breakages occurring in: ligand molecule, in the absence of Cu(II), ⇒; Cu(II)-amikacin complex ,--->; in both cases, →.

The DNA bases are not exposed in nucleic acids molecules. The attack on the guanine or any other nucleobases is then difficult due to tight coils of sugar-phosphate backbone. The radical species, especially diffusible hydroxyl radicals, usually react with the systems most proximal to their side of formation. So the primary damage in case of nucleic acids would occur at the backbone. This results in two different phenomena, single strand damage or the double one. The double strand damage may also be the consequence of accumulation of single strand cleavages, which then lead to further degradation to short DNA fragments /31, 32/. The stepwise character of plasmid DNA destruction through all the forms, caused by the complexes studied, suggests that the DNA fragmentation is a result of accumulation of random single strand breaks in the plasmid, rather than the immediate formation of double strand breaks. However, such a course was proposed for Cu(II) aqua ion interacting with DNA /33/. In fact, our control mixture of Cu(II) ions and $H_2O_2$ also produced double strand scission in a stepwise fashion, but with much lower rate and yield than the complex. From the dependence of plasmid DNA damage on pH, we conclude that the degradation starts at pH 5, where the first complex form appears in solution /32/ (Fig. 8). With the increase of pH the entire amount of the complex forms existing in solution also rises at the expense of the amount of uncomplexed copper(II). This brings growth of the linear form and causes the complete destruction of the superhelical one. At pH 7 and above, we observe almost total destruction of the plasmid. In this pH range the complex form, which is dominating in solution, is probably responsible for plasmid destruction.

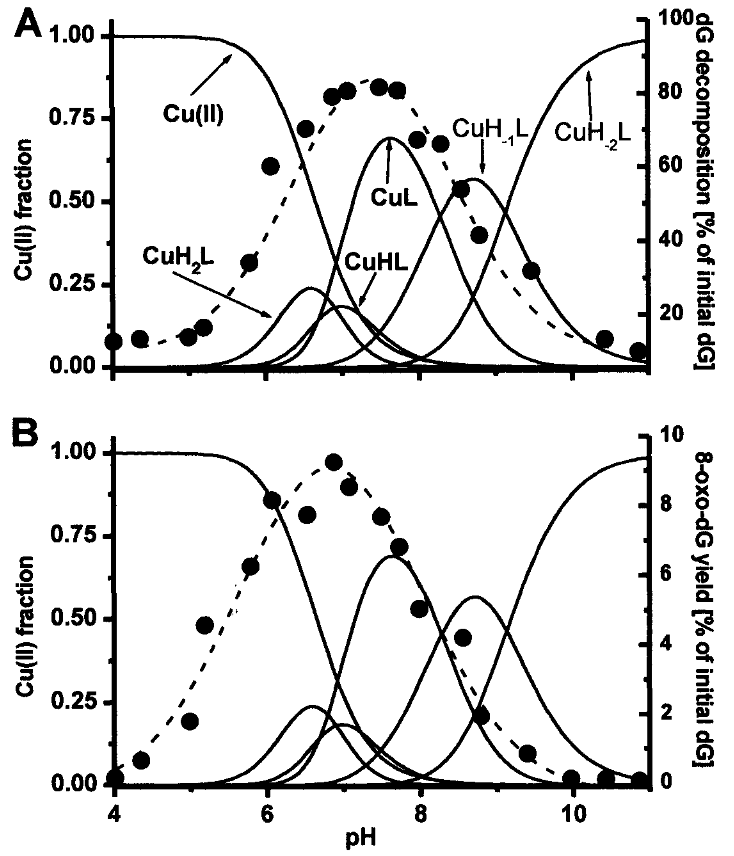

**Fig. 7:** The extent of dG destruction (A) and 8-oxo-dG formation (B) in the presence of Cu(II)-kanamycin A-$H_2O_2$ system, after 24 h incubation at 37 °C, in the pH range: 4 – 11, overlaid with species distribution diagram for Cu(II) – kanamycin A.

The copper aminoglycosides were shown recently to interact with HIV-1 viral Rev responsible element /34/ and *in vivo*, with R23 mRNA /35/. We have undertaken a study to describe the possibility of inducing the damage to tRNA by aminoglycosides and their cupric complexes. The cleavage occurring in the presence of antibiotic or the complex is highly specific and takes place in the anticodon loop, at hypermodified base – Y37 /30-32, 36/. Cleavage sites generated in the presence of copper-aminoglycosides in oxidative surroundings were more numerous. After the addition of Cu(II)-$H_2O_2$ system no breakage was noticed at Y37. This suggests that hypermodification present at this position is crucial for aminoglycoside binding and the hydrolysis. Moreover, this also seems to be a characteristic feature of these drugs.

Interestingly, the relative cleavage efficiency induced in the presence of Cu(II)-antibiotic complexes at Y37 strongly depended on the number of amino functions in the aminoglycoside molecules /30/. Figure 9 presents the dependence of the hydrolysis extents at Y37 on the resultant charge of Cu(II)-antibiotics complexes. It turns out that this correlation is linear. A possible explanation of this phenomenon is that the cleavage efficiency is a function of the complexes binding strength in the vicinity of Y37, which rises with the increase of the positive charge of the complexes.

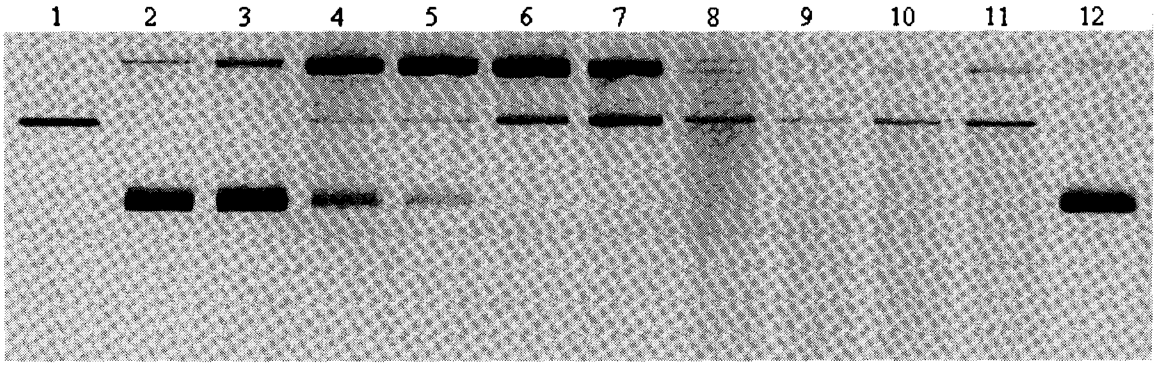

**Fig. 8:** pBluesriptSK+ plasmid DNA cleavage by Cu(II)-kanamycin A-$H_2O_2$ system in pH dependent mode. Lane 1, plasmid + *Eco*RI endonuclease; lane 2, plasmid at pH 4.5 (control); lane 3, pH 4.5; lane 4, pH 5.0; lane 5, pH 5.5; lane 6, pH 6.0; lane 7, pH 6.5; lane 8, pH 7.0; lane 9, pH 7.5; lane 10, pH 8.0; lane 11, pH 8.5; lane 12, control at pH 8.5.

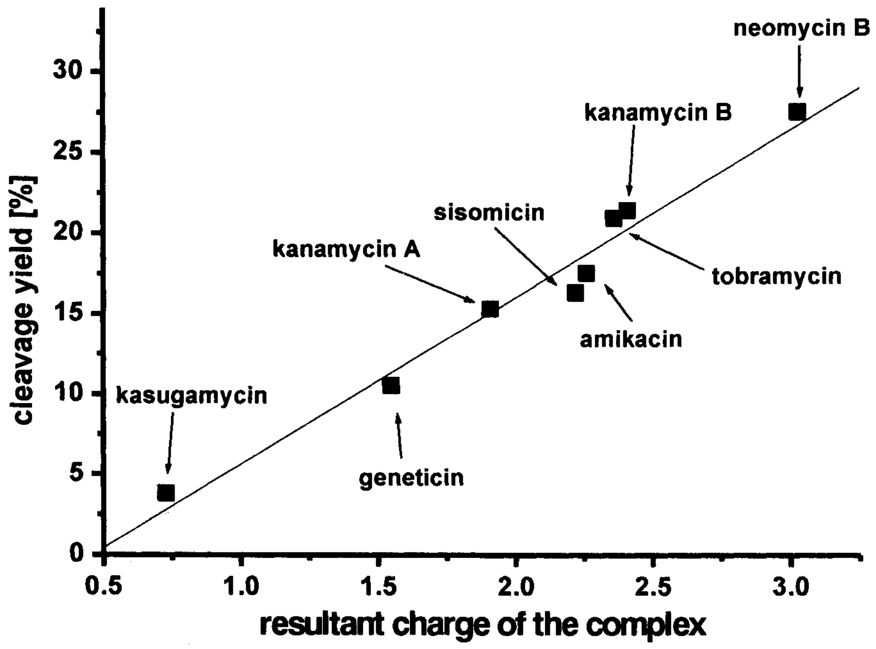

**Fig. 9:** Dependence of the extent of cleavage at Y37 on the resultant charge of Cu(II)-antibiotics complexes at pH 7.4.

In order to confirm the importance of hypermodification for the strand breakage process we compared susceptibility to the cleavage reaction of natural tRNA$^{Phe}$, its analogue devoid of modified nucleotides and acDNA – a DNA oligomer of tRNA anticodon stem sequence /30/. The results presented in Figure 10 clearly demonstrate that this cleavage occurred only within the natural tRNA$^{Phe}$. Thus the tRNA anticodon stem, neither in its RNA nor DNA form, is sufficient to undergo highly specific breakage. Hypermodified guanine residues occur in the anticodon loops of several tRNA$^{Phe}$ molecules, including human one. It is conceivable that those molecules may also undergo specific fragmentation in the presence of aminoglycoside antibiotics or their complexes.

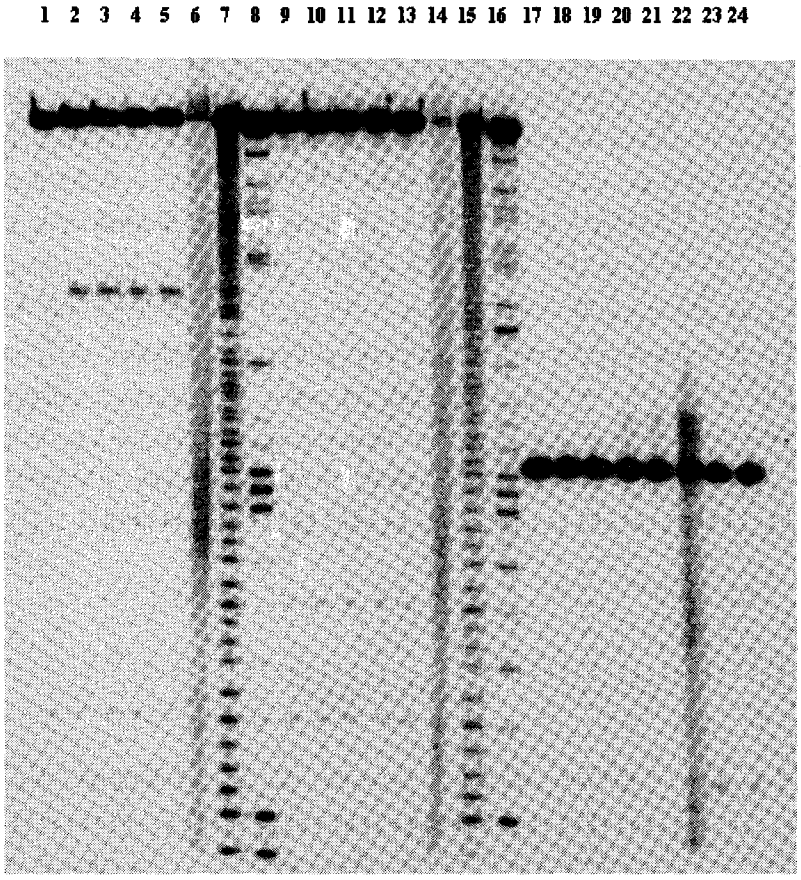

**Fig. 10:** Specificity of cleavages induced by aminoglycosides and their Cu(II) complexes within yeast tRNA$^{Phe}$ in comparison with unmodified tRNA$^{Phe}$ and DNA oligomer of anticodon loop sequence. Lanes 1-8, tRNA$^{Phe}$, lanes 9-17, unmodified tRNA$^{Phe}$, lanes 18-24, DNA with: lanes 1, 9, 17, untreated; lanes 2, 10, 18, + 50 μM kanamycin A; lanes 3, 11, 19, + 100 μM kanamycin A; lanes 4, 12, 20, + 50 μM kanamycin A + 50 μM $H_2O_2$; lanes 5, 13, 21, + 50 μM kanamycin A + 50 μM $CuCl_2$; lanes 6, 14, 22, + 50 μM kanamycin A + 50 μM $CuCl_2$ + 50 μM $H_2O_2$; lanes 7, 15, 23, formamide ladder; lanes 8, 16, 24, limited hydrolysis by RNase T1.

### BIOLOGICAL IMPLICATIONS

Recently we have also undertaken the study of microbiological activity of the complexes in comparison with the antibiotics alone, with use of 3 bacterial strains: *Pseudomonas aeruginosa, Staphylococus aureus* and *Escherichia coli.* The data indicate that copper ion does not influence the bactericidal efficacy of the antibiotics /32/. This may be the result of the complex hydrolysis in bacterial wall rich in lipopolysaccharides, which contain many aminosugar moieties, similar to those in antibiotics. As a consequence, copper may form stable complexes in the wall, but it requires further studies to estimate the transport of the complex through the bacterial cell wall.

Because antibacterial agents and metal ions have the possibility to stimulate immunity system resulting in generation of different factors /37, 38/, we examined the immunogenic activity of Cu(II)-aminoglycoside complexes by measuring their influence on human peripheral blood leukocytes. The results of our studies reveal that the complexes studied are non toxic for human lung adenocarcinoma cells at the concentrations occurring after injections of aminoglycoside antibiotics /39/. We have also found that low doses of the complexes may play an important role for the host, as stimulators of immune cells and phagocytes, by producing relatively high levels of IFN, TNF and IL-10 /17/. The effect on the cytokines release was dose and time depended and the interdependence between IL-10 and TNF stimulation was found. The ability of the cupric complexes of aminoglycosides to induce cytokines may enhance the production of further inflammatory agents by the cells. As a matter of fact, the effects of cytokines production by the complex stimulated leukocytes do not appear to be very high, however, any interference into the immunity system is disadvantageous and may enhance the known toxicity of aminoglycosides. The interactions of Cu(II)-antibiotics with the immune system may partially explain the undesirable effects of aminoglycosides on human tissues.

### CONCLUSIONS

The results of our studies indicate that aminoglycoside antibiotics effectively bind copper(II) ions at physiological pH. The complexes disproportionate $H_2O_2$ to dioxygen and water, in the process accompanied by both metal bound and diffusible hydroxyl radicals. These species have also impact on aminoglycosides-induced nucleic acids damage and nucleic bases oxidation. This clearly demonstrates that Cu(II) complexes of aminoglycosides may be regarded as potentially dangerous genotoxic agents and that the primary feature influencing their reactivity is the resulting charge of the complex. Although these species probably cannot enter bacterial cell wall, they activate the human tissue cells towards immunological mediators production. The facts presented herein may be significant for understanding the origins of the toxic effects induced by the drugs.

**ACKNOWLEDGEMENTS**

This work was supported by the Polish State Committee for Scientific Research (KBN), grants no. 4 T09A 02722 and 4 T09A 179 25.

**REFERENCES**

1. L. E. Bryan in: *New Dimensions in Antimicrobial Therapy*, R. K. Root, M. A. Sande (eds.), Churchill Livingstone Inc., New York, 1984.
2. A .Sreedhara, J.A.Cowan; *J. Biol. Inorg. Chem.*, **6**, 166 (2001).
3. J. Cho and R. R. Rando, *Biochemistry*, **38**, 8548 (1999).
4. B.R. Cullen, W.C. Green, *Cell*, **58**, 423 (1989).
5. U. von Ahsen, J. Davies, R. Schroeder, *J. Mol. Biol.*, **226**, 935 (1992).
6. T. K. Stage, K. J. Hertel and O. C. Uhlenbeck, *RNA*, **1**, 95 (1995).
7. N. E. Mikkelsen, M. Brannvall, A. Virtanen and L. Kirsebom, *Proc. Natl. Acad. Sci. USA*, **96**, 6155 (1999).
8. S. R. Kirk and Y. Tor, *Bioorg. Med. Chem.*, **7**, 1979 (1999).
9. B. D. Davis, *Microbiol. Rev.*, **51**, 341 (1987).
10. A. Forge, J. Schacht, *Audiol. Neurootol.*, **5**, 3 (2000).
11. S.-H. Sha, J. Schacht, *Hear. Res.*, **128**, 112 (1999).
12. B.-B. Song, S.-H. Sha, J. Schacht, *Free Rad. Biol. Med.*, **25**, 189 (1998).
13. B. J. Conlon, B. P. Perry, D. W. Smith, *Laryngoscope*, **108**, 284 (1998).
14. N. Barba-Behrens, J. L. Bautista, M. E. Ruiz, P. Joseph-Nathan, A. Flores-Parra, R. Contreras, *J. Inorg. Biochem.*, **40**, 201 (1990).
15. S. M. Abu-El-Wafa, M. A. El-Ries, F. M. Abou-Attia, R. M. Issa, *Anal. Lett.*, **22**, 2703 (1989).
16. Z. H. Chohan, M.-U. H. Ansari, *Pakistan J. Pharmacol.*, **6**, 21 (1989).
17. W. Szczepanik, A. Czarny, E. Zaczyńska and M. Jeżowska-Bojczuk, *J. Inorg. Biochem.*, submitted.
18. T. D. Rae, P. J. Schmidt, R. A. Pufahl, V. C. Culotta, T. V. O'Halloran, *Science*, **284**, 805 (1999).
19. M. C. Linder, *Biochemistry of Copper*, Plenum Press, New York 1991.
20. M. Jeżowska-Bojczuk, W. Bal and H. Kozłowski, *Inorg. Chim. Acta*, **275-276**, 541 (1998).
21. M. Jeżowska-Bojczuk, A. Karaczyn, W. Bal, *J. Inorg. Biochem.*, **71**, 129 (1998).
22. M. Jeżowska-Bojczuk, A. Karaczyn and H. Kozłowski, *Carbohydr. Res.*, **313**, 265 (1998).
23. W. Szczepanik, P. Kaczmarek, J. Sobczak, W. Bal, K. Gatner and M. Jeżowska-Bojczuk, *New J. Chem.*, **26**, 1507 (2002).
24. W. Leśniak, W. R. Harris, J. Y. Kravitz, J. Schacht, V. L. Pecoraro, *Inorg. Chem.*, **42**, 1420 (2003).
25. M. Jeżowska-Bojczuk, W. Bal, *J. Chem. Soc. Dalton Trans.*, 153 (1998).
26. K. Takahashi, O. Yoshioka, A. Matsuda, H. Umezawa; *J. Antibiot.*, **30**, 861 (1977).
27. G. M. Ehrenfeld, L. O. Rodriguez, S. M. Hecht, C. Chang, V. J. Basus, J. J. Oppenheimer, *Biochemistry*, **24**, 81 (1985).

28. M. Jeżowska-Bojczuk and W. Leśniak; *J. Inorg. Biochem.*, **85**, 99 (2001).

29. M. Jeżowska-Bojczuk, W. Leśniak, W. Bal, H. Kozłowski, K. Gatner, A. Jezierski, J. Sobczak, S. Mangani, W. Meyer-Klaucke, *Chem. Res. Toxicol.*, **14**, 1353 (2001).

30. W. Szczepanik, J. Ciesiołka. J. Wrzesiński, J. Skała and M. Jeżowska-Bojczuk, *J. Chem. Soc., Dalton Trans.*, 1488 (2003).

31. M. Jeżowska-Bojczuk, W. Szczepanik, W. Leśniak, J. Ciesiołka, J. Wrzesiński and W. Bal, *Eur. J. Biochem.*, **269**, 5547 (2002).

32. W. Szczepanik, E. Dworniczek, J. Ciesiołka. J. Wrzesiński, J. Skała and M. Jeżowska-Bojczuk, *J. Inorg. Biochem.*, **94**, 355 (2003).

33. S. Oikawa and S. Kawanishi, *Biochemistry,* **35**, 4584 (1996).

34. A. Sreedhara, J. A. Cowan, *J. Biol. Inorg. Chem.*, **6**, 166 (2001).

35. C.-A. Chen and J. A. Cowan, *Chem. Commun.*, 196 (2002).

36. S. R. Kirk and Y. Tor, *Bioorg. Med. Chem.*, **7**, 1979 (1999).

37. P. Griem, M. Wulferink, B. Sachs, J. B. Gonzalez and E. Gleichmann, *Immunol. Today*, **19**, 133 (1998).

38. E. Padovan, T. Bauer, M. M. Tongio, H. Kalbacher and H. U. Weltzien, *Eur. J. Immunol.*, **27**, 1303 (1997).

39. B. D. Davies, R. E. Brummett, T. W. Bendrick, and D. L. Himes, *J. Antimicrob. Chemother.*, **14**, 291 (1984).

